# The cognitive basis of social behavior: cognitive reflection overrides antisocial but not always prosocial motives

**DOI:** 10.3389/fnbeh.2015.00287

**Published:** 2015-11-05

**Authors:** Brice Corgnet, Antonio M. Espín, Roberto Hernán-González

**Affiliations:** ^1^Argyros School of Business and Economics, Economic Science Institute, Chapman UniversityOrange, CA, USA; ^2^Economics Department, Middlesex University Business SchoolLondon, UK; ^3^Granada Lab of Behavioral Economics, Universidad de GranadaGranada, Spain; ^4^Business School, University of NottinghamNottingham, UK

**Keywords:** dual-process, intuition, social preferences, altruism, spitefulness, prosocial behavior, antisocial behavior, inequality aversion

## Abstract

Even though human social behavior has received considerable scientific attention in the last decades, its cognitive underpinnings are still poorly understood. Applying a dual-process framework to the study of social preferences, we show in two studies that individuals with a more reflective/deliberative cognitive style, as measured by scores on the Cognitive Reflection Test (CRT), are more likely to make choices consistent with “mild” altruism in simple non-strategic decisions. Such choices increase social welfare by increasing the other person's payoff at very low or no cost for the individual. The choices of less reflective individuals (i.e., those who rely more heavily on intuition), on the other hand, are more likely to be associated with either egalitarian or spiteful motives. We also identify a negative link between reflection and choices characterized by “strong” altruism, but this result holds only in Study 2. Moreover, we provide evidence that the relationship between social preferences and CRT scores is not driven by general intelligence. We discuss how our results can reconcile some previous conflicting findings on the cognitive basis of social behavior.

## Introduction

Mounting evidence shows that humans cooperate with non-kin even when doing so implies paying irrecoverable costs (Ledyard, 1995; Gintis, 2000; Henrich et al., 2001; Fehr and Gächter, 2002; Bowles and Gintis, 2003; Camerer, 2003). These prosocial behaviors are inconsistent with the strict pursue of self-interest and thus constitute a challenge for disciplines ranging from evolutionary biology to the social and behavioral sciences (Nowak, 2006; Fehr and Camerer, 2007; Harbaugh et al., 2007; Perc and Szolnoki, 2008; Roca et al., 2009; Capraro, 2013; Exadaktylos et al., 2013; Rand and Nowak, 2013; Gutiérrez-Roig et al., 2014; Raihani, 2014).

In recent years, the cognitive underpinnings of social behavior have been increasingly studied, as their understanding is key for building a comprehensive account of the proximate—and, indirectly, also ultimate—explanations of human sociality (Stevens and Hauser, 2004; Rand and Nowak, 2013; Zaki and Mitchell, 2013). Much of the advances on this front have been made within the framework of dual-process theories, which point to the existence of an interaction between fast, automatic/intuitive (“System 1”) and slow, controlled/reflective (“System 2”) decision making processes (Hogarth, 2001; Stanovich, 2010; Kahneman, 2011). From this perspective, most research has focused on answering the question of whether human prosocial (as opposed to selfish) behavior is the result of intuition or reflection (Loewenstein and O'Donoghue, 2004; Moore and Loewenstein, 2004; Rand et al., 2012; Zaki and Mitchell, 2013). In other words, are humans' automatic responses selfish or prosocial?

An extensive research program on the topic has identified cooperation as the intuitive response in anonymous one-shot social dilemma experiments, with further reflection leading to more selfish choices (Rand et al., 2012, 2014, 2015; Cone and Rand, 2014; Rand and Kraft-Todd, 2014; Evans et al., 2015). These findings have led to the Social Heuristics Hypothesis (SHH; Rand et al., 2014), according to which people internalize social behaviors that generate personal benefits in daily life. In contrast to most economic experiments, daily life interactions are often repeated and face-to-face, and this implies that behaving cooperatively may be rewarding in the long run (through reciprocity, reputation or due to the existence of sanctions; Hamilton, 1964; Williams, 1966; Trivers, 1971; Fudenberg and Maskin, 1986; Bowles and Gintis, 2003). Individuals interacting in environments where helping others usually pays off would thus be more likely to internalize prosocial behaviors than individuals dwelling more “inhospitable” environments (Rand et al., 2012; Peysakhovich and Rand, 2015). Such internalization would lead people to apply prosocial heuristics even in situations where cooperation is maladaptive, such as in one-shot anonymous economic experiments.

Even though the SHH has received considerable empirical support (Roch et al., 2000; Cornelissen et al., 2011; Rand et al., 2012, 2014, 2015; Lotito et al., 2013; Cone and Rand, 2014; Nielsen et al., 2014; Rand and Kraft-Todd, 2014; Schulz et al., 2014; Capraro and Cococcioni, 2015; Evans et al., 2015), a number of findings seem inconsistent with the idea of spontaneous prosociality and calculated selfishness (e.g., Knoch et al., 2006, 2010; Dewall et al., 2008; Piovesan and Wengström, 2009; Martinsson et al., 2012, 2014; Xu et al., 2012; Tinghög et al., 2013; Crockett et al., 2014; Jaber-López et al., 2014; Verkoeijen and Bouwmeester, 2014). In this paper, we shall argue that our understanding of the sources of these apparent contradictions may benefit from an in-depth analysis of the motivations underlying social behavior. A distinction should thus be made between observed behavioral outcomes and underlying social motivations (Falk et al., 2005; Jensen, 2010; Espín et al., 2012; Brañas-Garza et al., 2014). Indeed, a variety of “prosocial” motivations (e.g., altruism or egalitarianism; see below) can trigger seemingly identical prosocial behaviors. It might be the case that some of the prosocial motivations that account for a specific behavior are linked to intuition whereas others are linked to reflection. This may explain why the analysis of isolated social decisions has led to mixed findings regarding the role of intuitive and reflective processes in prosocial behavior.

The previous discussion focused on the often-studied prosocial side of human behavior but it nonetheless extends to the less-studied antisocial side. Evidence from economic experiments also shows that people often make “antisocial” decisions that reduce others' welfare without any apparent personal gain (Zizzo and Oswald, 2001; Fehr and Gächter, 2002; Knoch et al., 2006; Herrmann and Orzen, 2008; Herrmann et al., 2008; Abbink et al., 2010; Espín et al., 2012; Kimbrough and Reiss, 2012; Brañas-Garza et al., 2014). Spiteful behaviors that harm others even at one's own cost may yet be advantageous, for example, in social environments where survival hinges upon one's relative standing in the group^1^. Therefore, following the SHH argument, some people might internalize behaviors that not only promote but also reduce others' welfare as an adaptation to their daily life interactions. Welfare-reducing behaviors are likely to respond to antisocial motives that aim at increasing one's relative standing (Kirchsteiger, 1994; Van Lange, 1999; Charness and Rabin, 2002; Jensen, 2012). This logic has been applied, for instance, to understanding the punishment decisions of non-cooperators in social dilemma games (Shinada et al., 2004; Falk et al., 2005; Gächter and Herrmann, 2011; Espín et al., 2012). When the punishing individual is a cooperator, however, fairness-based explanations are often put forward (Fehr and Schmidt, 1999; Fehr and Gächter, 2002; Gächter and Herrmann, 2009; Espín et al., 2012). From this viewpoint, fairness concerns, which are traditionally considered to be prosocial (Van Lange, 1999), can also lead to behaviors that reduce the payoff of another individual.

To analyze the cognitive underpinnings of human social interaction, we believe it is important to distinguish people's actual behaviors and motivations. To do so, it is necessary to bring back the too-often ignored antisocial motivations at the center of the debate. Our research thus aims at studying a broad range of prosocial as well as antisocial motives and assess to which extent these motives are driven by either intuition or reflection.

### Disentangling social motives

To assess the motivations behind social decisions, we consider an “outcome-based”—or distributional—social preferences model, namely the inequality-aversion model of Fehr and Schmidt (1999), which introduces the payoffs of relevant others into the individuals' utility function. Individuals with outcome-based social preferences behave *as if* they were maximizing a utility function which includes a concern for the payoff of others, in addition to their own payoff. In particular, Fehr and Schmidt (1999) account for a potential asymmetry between advantageous and disadvantageous payoff comparisons between the self and a referent other (e.g., Loewenstein et al., 1989). We extend the previous model so as to capture behaviors that may not strictly follow from standard inequality-aversion preferences. We will rely on a generalized and flexible specification of preferences that will allow us to disentangle competing explanations of individuals' decisions, including both prosocial and antisocial motivations. Similar approaches have been followed for instance by Charness and Rabin (2002); Engelmann and Strobel (2004); Engelmann (2012) and Cox (2013)^2^.

As mentioned, one caveat in the categorization of social behavior is that individuals' decisions in standard economic games are typically consistent with different types of motivations. For instance, both spiteful and selfish motives would identically lead to zero transfers in dictator games (Brañas-Garza et al., 2014) or to defection in social dilemma games (Falk et al., 2005; Espín et al., 2012). Similarly, the acceptance of a low offer in the ultimatum game could result from either selfishness or altruism (Staffiero et al., 2013). In order to uncover the driving forces behind a particular decision, a clear cut procedure is to observe the decisions made by the same individual in different social situations (Falk et al., 2005; Espín et al., 2012; Yamagishi et al., 2012; Staffiero et al., 2013; Brañas-Garza et al., 2014; Peysakhovich et al., 2014). In addition, these decisions should be free of strategic or reciprocal concerns since these could alter behavior and distort the assessment of outcome-based preferences (Charness and Rabin, 2002). Building on this argument, our experimental design makes use of several decisions in short, cognitively undemanding and non-strategic tasks.

### A trait approach to cognitive reflection

To isolate intuitive and reflective cognitive processes, previous behavioral research on social behavior has primarily relied on the analysis of reaction times (e.g., Rubinstein, 2007; Piovesan and Wengström, 2009; Brañas-Garza et al., 2012b; Rand et al., 2012; Lotito et al., 2013) and the use of experimental manipulations, such as cognitive load (e.g., Cornelissen et al., 2011; Duffy and Smith, 2014; Hauge et al., 2014; Schulz et al., 2014) or time pressure (e.g., Tinghög et al., 2013; Cone and Rand, 2014; Rand et al., 2014, 2015; Rand and Kraft-Todd, 2014). In this paper, we adopt a trait approach which relies on the assumption that individuals who have a more intuitive cognitive style are more likely to make decisions guided by automatic processes (System 1), whereas more reflective individuals are more likely driven by deliberative processes (System 2) (Oechssler et al., 2009; Toplak et al., 2011; Peysakhovich and Rand, 2015). Subjects' cognitive styles are assessed through the Cognitive Reflection Test (CRT; Frederick, 2005), which measures the ability to override intuitive responses and to engage in further reflection before making a decision. The CRT is a short task consisting of a set of insights problems (three in the original form of Frederick, 2005; and seven in the extended version introduced by Toplak et al., 2014). The CRT differs from other measures of cognitive abilities as it is designed to prompt an intuitive, yet incorrect, answer to the respondent's mind. To reach the correct answer, the person must override this automatic response by engaging in reflection.

The CRT fits in nicely with the dual-process approach of decision making. The responses to the test are indeed a good proxy for the individuals' tendency to make intuitive vs. reflective decisions. CRT scores have been found to predict one's own ability to refrain from using inaccurate heuristics in a variety of situations (Oechssler et al., 2009; Toplak et al., 2011)^3^. Furthermore, there is evidence that the same behaviors that are observed after experimental manipulations of intuitive processing covary with CRT scores in the expected direction (e.g., Shenhav et al., 2012). With regards to social behavior, Peysakhovich and Rand (2015) show that an individual's score on the CRT can predict her tendency to apply previously-acquired social heuristics in environments where they are not advantageous. The authors first conducted repeated social dilemmas where cooperation was or was not advantageous before embedding subjects in one-shot games (social dilemma, dictator, and trust games) where prosocial behavior was detrimental to subjects' payoff. As predicted by the SHH, subjects who had interacted in the environment where cooperation was advantageous were on average more prosocial in the subsequent one-shot games compared to those who had interacted in the environment where cooperation was disadvantageous. However, after separating subjects according to cognitive style, the authors show that the predicted spillover effect was only observed among subjects with low CRT scores.

Our empirical strategy will be to correlate subjects' answers to the extended version of the CRT (Toplak et al., 2014) with their decisions in the social preferences elicitation task. A similar approach has been undertaken in an independent study conducted by Cueva et al. (in press) and Ponti and Rodriguez-Lara (2015). We present the results of two studies one of which was conducted in the US and the other in Spain.

## Study 1

### Methods

#### Participants and general protocol

Participants were 150 students [44.67% female; mean age 20.61 ± 2.73 (SD)] from Chapman University in the U.S. Participants were enrolled in the following majors at the time of the study: Business and Economics (28.7%), Humanities and Social Sciences (21.3%), Science and Technology (15.3%), Film Studies (16.7%), Performing Arts (4.0%), Health and Behavioral Sciences (4.0%), Law School (2.7%), Educational Studies (2.0%) or other studies (5.3%). These participants were recruited from a database of more than 2000 students. A subset of the whole database received invitations at random for participating in the current study, which is part of a larger research program on cognitive abilities and economic decision making. The local IRB approved this research. All participants provided informed consent prior to participating. No deception was used.

We conducted a total of 12 sessions, nine of which with 12 participants and three of which with 14 participants. On average, sessions lasted for 45 min. All subjects completed the same tasks in the same order given that we would need a much larger sample size in order to statistically control for the effect of all possible task sequences. The order and nature of the tasks are shown in (Supplementary) Text S1. Importantly, since our aim is to study reflection as a cognitive disposition (i.e., the trait approach), the social preferences elicitation task was performed before the CRT. Otherwise, having completed the CRT could have induced a reflective mindset which might alter the relationship between trait reflectiveness and the behavior under study (Paxton et al., 2012). In any case, in between the social preferences elicitation task and the CRT participants completed a series of unrelated tasks for about 15 min and had a break of 10 min to reduce the potential influence of exhaustion or cognitive load. This protocol also alleviates concerns about the existence of between-tasks spillover effects (e.g., Fromell et al., 2014) which may potentially induce reverse causality. However, none of these two factors can be completely ruled out with our procedure and concerns about the influence of uncontrolled variables remain.

#### Cognitive style assessment

We measured the participants' tendency to rely on intuition vs. reflection using the Cognitive Reflection Test introduced by Frederick (2005). To the original CRT questions, we added four questions recently developed by Toplak et al. (2014). The full set of questions can be found in (Supplementary) Text S2. In Table S1, we display the % of subjects answering each question correctly, split by gender. As expected, males performed better in the test than females (Frederick, 2005; Bosch-Domènech et al., 2014) and this difference was statistically significant (see Table S1). Our measure of cognitive reflection is given by the total number of correct answers (from 0 to 7). The full distribution of correct answers by males (mean = 3.67 ± 2.25) and females (mean = 2.39 ± 1.95) is provided in Figure S1.

In addition to CRT, we also measured general intelligence which is likely to be a confounding factor of the (potential) relationship between CRT scores and social behavior. Because answering CRT questions require cognitive abilities, CRT scores partly capture general intelligence in addition to cognitive reflection (Frederick, 2005; Stanovich, 2009). However, cognitive reflection differs from intelligence as measured in standard IQ tests (e.g., Raven matrices). Intelligence tests measure one's capacity to compute solutions to problems but fail to assess one's capacity to engage in reflection (Stanovich, 2009). Although basic cognitive abilities are required to answer the CRT correctly, an intelligent person may often rely on automatic answers (System 1) falling short of blocking intuitive processes by engaging in reflection (System 2). In order to evaluate the importance of general intelligence as a possible confound in the relationship between CRT and social behavior, we measured subjects' IQ using the Raven progressive matrices test (Raven, 1941) and used it as a control variable in our analyses. Specifically, we used the odd number of the last three series of matrices (Jaeggi et al., 2010). The number of matrices correctly solved in the Raven test (in our sample, ranging from 8 to 18, mean = 14.61 ± 2.12) is a conventional measure of cognitive ability. This test captures an important aspect of cognitive ability which is referred to as fluid intelligence or algorithmic thinking (Stanovich, 2009, 2010).

Consistently with Frederick (2005) and Stanovich (2009, 2010) we find moderate positive correlation between the number of correct answers in the CRT and Raven tests (*r* = 0.43, *p* < 0.01) which suggests that CRT and Raven are not entirely measuring the same cognitive skills. As is standard practice, none of the cognitive tests were incentivized (Frederick, 2005).

#### Social preferences elicitation

We elicited social preferences à la Bartling et al. (2009) by asking participants to make four choices between two possible allocations of money between themselves and another anonymous participant with whom they were randomly matched. All participants made all the four decisions. We used this short task because it provides a good balance between (maximizing) the information that can be obtained and (minimizing) the cognitive effort required to complete the task. In each experimental session, two participants and one of the four decisions were selected at random for payment. The choice of the first participant in the selected decision was used to allocate payoffs between the two participants (e.g., Sheremeta and Shields, 2013). All decisions were anonymous.

The allocation decisions are described in Table 1. Option A always yielded an even distribution of money ($2 for both the self and the other participant), whereas option B yielded uneven payoffs. The first two decisions refer to the advantageous domain while the last two decisions refer to the disadvantageous domain. For each decision, we show in parentheses the envy/compassion parameter associated to choosing the egalitarian and non-egalitarian options (i.e., options A and B) and in square brackets the proportion of subjects who chose each option. In order to compute the model parameters, we assume that utility is linear over the range of payoffs involved in the task (Fehr and Schmidt, 1999). According to the basic specification of the model (Fehr and Schmidt, 1999) for the two-person case, the utility derived by individual *i* from the payoff vector *X* = (*x*_*i*_, *x*_*j*_) is given by:

(1) $$\begin{array}{r} U_{i}\left(X\right)=x_{i}-\alpha_{i}max\;\left\{x_{j}-x_{i},0\right\}-\beta_{i}max\;\left\{x_{i}-x_{j},0\right\} \end{array}$$

where the parameters α_*i*_ and β_*i*_ refer to the individual *i*'s *aversion* to disadvantageous (i.e., “envy”) and advantageous inequality (i.e., “compassion”), respectively. Thus, a self-regarding individual who is indifferent to others' payoffs would exhibit α_*i*_ = β_*i*_ = 0. A person with other-regarding motives would prefer either to increase or decrease others' payoffs depending on the sign and value of α_*i*_ [β_*i*_] if others' payoffs are above [below] her own payoffs.

**Table 1:** **Decisions in the social preferences task (Study 1)**.

**Decision #**	**Option A** **self, other**		**Option B** **self, other**	
1	$2,$2	(β ≥ 0) [86%]	$2,$1	(β ≤ 0) [14%]
2	$2,$2	(β ≥ 0.5) [23%]	$3,$1	(β ≤ 0.5) [77%]
3	$2,$2	(α ≥ 0) [42%]	$2,$4	(α ≤ 0) [58%]
4	$2,$2	(α ≥ 0.5) [31%]	$3,$5	(α ≤ 0.5) [69%]

For each option, we display the payoff for the decision-maker and the recipient, the associated model parameters (in parentheses) and the % of subjects choosing it (in square brackets). N = 150.

Fehr and Schmidt (1999) assume α_*i*_ ≥ β_*i*_ ≥ 0, which means that individuals can be either egalitarian (α_*i*_ ≥ 0 and β_*i*_ ≥ 0; with at least one inequality being strict) or selfish (α_*i*_ = β_*i*_ = 0). This parameterization also implies that people are assumed to display at least as envy as compassion (α_*i*_ ≥ β_*i*_). We do not impose these restrictions on the model parameters so that individuals' motivations can be characterized as follows:

- *Self-interest* if individuals' decisions maximize their own payoff (α_*i*_ = 0 and β_*i*_ = 0);
- *Altruism* if individuals' decisions maximize the other's payoff (α_*i*_ ≤ 0 and β_*i*_ ≥ 0; with at least one inequality being strict)—a concern for *social welfare* also applies if, in addition, |α_*i*_|, |β_*i*_| < 0.5 (Engelmann, 2012)^4^ —;
- *Egalitarianism* if individuals' decisions minimize payoff inequality (α_*i*_ ≥ 0 and β_*i*_ ≥ 0; with at least one inequality being strict);
- *Spitefulness* if individuals' decisions minimize the other's payoff (α_*i*_ ≥ 0 and β_*i*_ ≤ 0; with at least one inequality being strict)—which, for empirically relevant values of α_*i*_ and β_*i*_, also implies a preference for increasing the individual's relative standing;
- *Inequality-seeking* if individuals' decisions maximize payoff inequality (α_*i*_ ≤ 0 and β_*i*_ ≤ 0; with at least one inequality being strict)—note that we include this type of preferences for the sake of completeness even though few individuals typically fall into this category.

Hence, we classify individuals' motives according to the combination of both model parameters. Following previous literature, we shall consider that altruism and egalitarianism are prosocial preferences (e.g., Van Lange, 1999; Fehr and Schmidt, 2006) while spitefulness is antisocial (e.g., Herrmann and Orzen, 2008; Jensen, 2012; Brañas-Garza et al., 2014).

As it happens with nearly every single decision in social interactions, each choice is consistent with multiple social preferences. For instance, in Decision 1 the participants had to decide whether or not to increase the payoff of a worse-off counterpart by $1 at no cost—or, alternatively, whether or not to reduce the other's payoff below one's own by $1 at no cost. Choosing option A in Decision 1 implies β ≥ 0 (compassion) and thus it may, depending on the exact value of β and the sign of α, be consistent with either egalitarianism, altruism, social-welfare concerns or self-interest (a selfish individual would choose randomly in this decision). Option B in Decision 1 is associated with β ≤ 0, which means that it can be chosen by individuals driven by either spitefulness or self-interest. Note that Decision 2 resembles the standard dictator game (Forsythe et al., 1994) in the sense that increasing the other's payoff does not increase the total surplus, i.e., social welfare. On the other hand, Decisions 3 and 4 resemble the decision of a second player (responder) in the standard ultimatum game (Güth et al., 1982)—if we leave reciprocal concerns aside—who has to choose whether to reject (option A) or accept (option B) a disadvantageous split proposed by the first player (proposer).

## Results and discussion

### Decision analysis

In Table 2, we report the results of Probit models estimating the likelihood of choosing option B (i.e., the non-egalitarian choice) in each of the four decisions as a function of CRT scores, Raven scores and gender. Note that a Bonferroni-like correction for multiple comparisons is not appropriate in this case because the decisions are substantially correlated as they all help measure social preferences (through estimates of different intervals for the (α, β) parameters) (see Table S3). To alleviate concerns about multiple comparisons, we present a multivariate Probit analysis in Table S5 and show that the results are remarkably similar.

**Table 2:** **Non-egalitarian choice (option B) as a function of CRT and Raven (Study 1)**.

	**Decision 1**	**Decision 2**	**Decision 3**	**Decision 4**
**Dep var:**	**β ≤ 0 (vs ≥ 0)**	**β ≤ 0.5 (vs ≥ 0.5)**	**α ≤ 0 (vs ≥ 0)**	**α ≤ 0.5 (vs ≥ 0.5)**
	**(1a)**	**(2a)**	**(3a)**	**(4a)**
CRT	−0.136^**^	−0.054	0.249^***^	0.236^***^
	(0.065)	(0.054)	(0.055)	(0.059)
	[−0.029^**^]	[−0.016]	[0.083^***^]	[0.073^***^]
Female	−0.573^*^	−0.329	−0.205	−0.219
	(0.306)	(0.236)	(0.224)	(0.233)
	[−0.120^**^]	[−0.098]	[−0.069]	[−0.068]
Cons	−0.465^*^	1.077^***^	−0.438^*^	−0.070
	(0.278)	(0.252)	(0.230)	(0.233)
	[0.321]	[0.859]	[0.331]	[0.472]
ll	−57.017	−79.185	−88.038	−81.547
Wald χ^2^	5.27^*^	2.43	23.72^***^	19.25^***^
Pseudo R^2^	0.061	0.014	0.137	0.126
	**(1b)**	**(2b)**	**(3b)**	**(4b)**
Raven	−0.061	0.034	0.093^*^	0.121^**^
	(0.052)	(0.055)	(0.050)	(0.050)
	[−0.013]	[0.010]	[0.035^*^]	[0.040^**^]
Female	−0.438	−0.242	−0.468^**^	−0.446^**^
	(0.273)	(0.228)	(0.211)	(0.219)
	[−0.095]	[−0.072]	[−0.175^*^]	[−0.149^**^]
Cons	−0.024	0.373	−0.945	−1.051
	(0.852)	(0.811)	(0.745)	(0.742)
	[0.490]	[0.645]	[0.172]	[0.147]
ll	−58.909	−79.473	−97.676	−88.273
Wald χ^2^	3.25	1.45	8.33^**^	9.50^***^
Pseudo R^2^	0.030	0.010	0.043	0.054
	**(1c)**	**(2c)**	**(3c)**	**(4c)**
CRT	−0.134^*^	−0.090	0.253^***^	0.221^***^
	(0.075)	(0.057)	(0.061)	(0.064)
	[−0.028^*^]	[−0.026]	[0.084^***^]	[0.068^***^]
Raven	−0.007	0.074	−0.009	0.036
	(0.070)	(0.059)	(0.055)	(0.055)
	[−0.001]	[0.022]	[−0.003]	[0.011]
Female	−0.571^*^	−0.361	−0.202	−0.233
	(0.309)	(0.234)	(0.226)	(0.236)
	[−0.120^*^]	[−0.106]	[−0.067]	[−0.072]
Cons	−0.376	0.131	−0.319	−0.540
	(0.936)	(0.817)	(0.734)	(0.749)
	[0.353]	[0.552]	[0.375]	[0.304]
ll	−57.012	−78.420	−88.026	−81.365
Wald χ^2^	5.41	4.57	23.72^***^	19.30^***^
Pseudo R^2^	0.061	0.023	0.137	0.128
N	150	150	150	150

Probit estimates. The α and β parameters associated with the dependent variable are displayed on top of each column. In “a” regressions, the main explanatory variable is CRT score. In “b” regressions, the main explanatory variable is Raven score. In “c” regressions, both CRT and Raven scores are included as explanatory variables. Robust standard errors clustered on individuals are shown in parentheses and average marginal effects of the explanatory variables are shown in square brackets (for the constant, this value represents the probability obtained from normal transformation of the Probit coefficient).

*, **, *** denote p-values lower than 0.10, 0.05, and 0.01, respectively.

**Figure 1:**
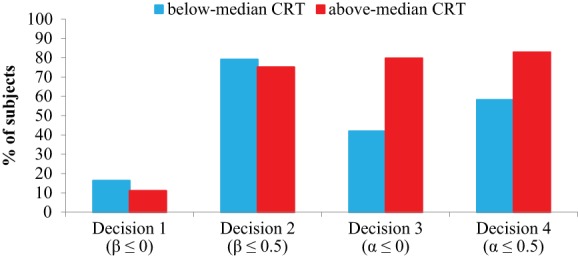
**Percentage of subjects choosing option B in each decision, by CRT groups (Study 1)**. The model parameters associated to option B are shown in parentheses.

Models in columns (1a)–(4a) of Table 2 estimate the likelihood of choosing option B as a function of CRT scores, and controlling for gender. Columns (1b)–(4b) replicate the same regressions but using Raven scores, instead of CRT, as the main explanatory variable. Finally, in columns (1c)–(4c) both CRT and Raven are included as regressors. Robust standard errors clustered at the individual level are presented in parentheses and marginal effects are presented in square brackets. In Figure 1 we display the % of subjects choosing option B in each decision, broken down into two CRT groups, namely individuals with below-median (i.e., three or less correct answers, *n* = 86) and above-median (*n* = 64) scores.

From column (1a) of Table 2, we observe that the CRT score is negatively and significantly associated with the choice of option B in Decision 1 (*p* = 0.04), suggesting that more reflective subjects are less likely to reduce the counterpart's payoff below their own payoff. In terms of the model parameters, subjects with higher CRTs are less likely to exhibit β ≤ 0. A two-sided binomial test rejects the hypothesis that above-median CRTs are indifferent between the two options in Decision 1 (i.e., 50% probability of choosing option B, *p* < 0.01), as would be the case for an individual motivated by self-interest (i.e., β = 0). For below-median CRTs, the binomial test yields a similar result (*p* < 0.01). Therefore, regardless of CRT, most subjects seem to exhibit strictly positive compassion (β>0) (see Figure 1). The marginal effect of CRT scores on Decision 1 is −0.029 (Table 2, column 1a), which means that the dependent variable changes by 2.9% for each 1-point increase in CRT scores. Since CRT ranges between 0 and 7, the difference between CRT = 0 and CRT = 7 in terms of the dependent variable is about 20%. As can be seen in Table 1, the mean proportion of non-egalitarian choice in Decision 1 is 14%, so that the predicted probability of choosing the non-egalitarian option in Decision 1, on average and roughly speaking (as it depends also on gender), goes from 24% for CRT = 0 to 4% for CRT = 7.

However, in Decision 2, where increasing the other's payoff is costly, CRT is no longer significant (*p* = 0.30, column 2a). This result suggests that the probability that the compassion parameter exceeds 0.5 does not differ across CRT scores. Additionally, within both the above-median and below-median CRT groups, a two-sided binomial test rejects that subjects are indifferent between the two options (ps < 0.01). This suggests that, regardless of CRT, β≠0.5. Indeed, for both above- and below-median CRTs, the % of subjects choosing option B is strictly above 50%, suggesting a median β strictly below 0.5 (see Figure 1).

Taken together, the results of Decision 1 and 2 indicate that, whereas the majority of subjects exhibit βϵ(0, 0.5), subjects with lower CRT scores are yet significantly more likely to exhibit β ≤ 0.

With respect to disadvantageous comparisons, column (3a) shows that CRT positively and significantly predicts choosing option B in Decision 3 (*p* < 0.01), which indicates that more reflective individuals are more likely to exhibit α ≤ 0. From Figure 1, we observe that this effect is strong, as nearly 80% of the subjects with above-median CRT decide not to lower their counterpart's payoff (this is significantly different from 50%: two-sided binomial test, *p* < 0.01), while only about 42% of below-median CRTs do so (which is not significantly different from 50%, *p* = 0.16). These results suggest that high-CRT individuals are not indifferent between both options in Decision 3—as would be the case for an individual motivated by self-interest, i.e., α = 0. In sum, high-CRT individuals are mostly characterized by α < 0, while the envy parameter that best characterizes low-CRT individuals seems to be close to zero or even slightly positive.

The results for Decision 4 are similar to those for Decision 3 as option B is positively and significantly predicted by CRT (*p* < 0.01, column 4a). This suggests that more reflective individuals are also more likely to exhibit α ≤ 0.5. Observing that more than 80% of the above-median CRT subjects choose option B in Decision 4 (see Figure 1; this proportion is significantly different from 50%: two-sided binomial test, *p* < 0.01), we can conclude that the envy parameter that best describes high CRTs is strictly lower than 0.5. In the case of below-median CRTs, however, this percentage falls to 58% (which is not significantly different from 50%, *p* = 0.16). Following the results of Decisions 3 and 4, low-CRT subjects, on average, display values of α which are apparently higher than those of high-CRT subjects.

Note that the qualitative nature of our statistical results does not depend on whether we use CRT scores or a binary categorization of CRT (as in Figure 1). Using above-median (vs. below-median) CRT as a binary explanatory variable in the regression analysis instead of CRT scores yields similar results (see Table S7). The effect of CRT in Decision 1 is, however, no longer significant at standard levels (*p* = 0.21).

Now, we turn to the second set of regressions of Table 2 (columns 1b–4b), where subjects' choices are estimated as a function of Raven scores. For those decisions for which CRT was found to be a significant predictor (namely Decisions 1, 3, and 4), the effect of Raven is qualitatively similar to that of CRT, although it seems to be less important (even non-significant in the case of Decision 1, *p* = 0.28). These results may indicate that a non-negligible share of the observed relationship between CRT and social preferences is actually driven by general intelligence. In order to address this point, we conducted a last series of regressions in which the scores on both cognitive measures are included as explanatory variables (columns 1c–4c). The regression results point to the opposite direction: the effect of CRT remains statistically significant while the significance of Raven scores completely vanishes when both variables are included in the same model. Note that this effect cannot be attributed to collinearity issues. A quick comparison of the regressions displayed in panel (a) and (c) shows that the standard error of the coefficient associated to the Raven variable increases only very slightly. More formally, using standard collinearity diagnostic analysis for all the regressors used in panel (c) regressions we report variance inflation factors for CRT, Raven and gender of 1.24, 1.14, and 1.09 which indicates the absence of collinearity problems^5^. Thus, CRT is a more important determinant of social preferences than Raven. Note that the coefficient associated to Raven scores is reduced by more than 70% after controlling for CRT in the three aforementioned decisions. Given that CRT accounts for virtually all the effect of Raven on social decisions, we can conclude that general intelligence is not confounding the relationship between CRT and social preferences. For our subsequent analysis we will thus focus on the analysis of CRT scores.

### Social preferences categorization

According to the above results, the decisions of most high-CRT individuals can be characterized as non-envious, i.e., α < 0, and moderately compassionate, i.e., β *ϵ* (0, 0.5). Although the majority of low-CRT individuals seem to be moderately compassionate as well they differ from high-CRT individuals by being envious. In addition, individuals with lower CRT scores are also significantly more likely to exhibit a non-positive compassion parameter (β ≤ 0), which in combination with envy (α>0) would be a sign of *antisocial*, spiteful motivations. As previously argued, combining both α and β is essential to obtain a complete picture of the motives driving social behavior. Our next analyses address this point.

**Figure 2:**
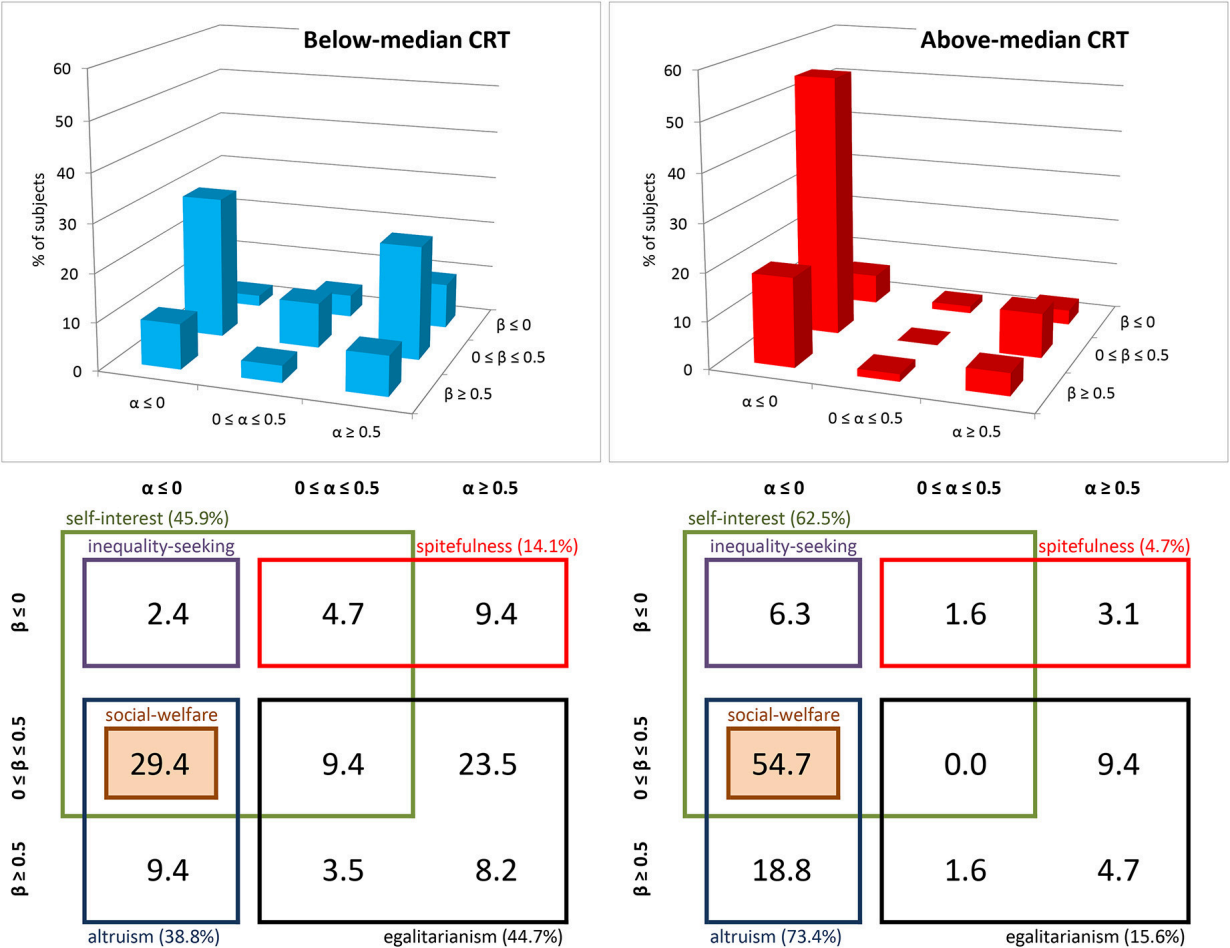
**Classification of subjects according to the envy and compassion parameters, by CRT groups (Study 1)**. The figure displays the % of subjects that can be classified according to each combination of α and β and the social preferences which are consistent with each category, broken down into below-median (*n* = 85) and above-median (*n* = 64) CRT score groups.

Figure 2 displays the % of individuals who are classified according to all possible combinations of the α and β parameters. Note that we include only those subjects with consistent choices, that is, choices which lead to compatible estimates of both α and β. This procedure excludes only one subject (out of 150). The left and right panels refer to subjects with below- and above-median CRT scores. In the table below each 3D plot, we highlight which among the combinations of the α and β parameters are consistent with each of the six categories of social motives previously defined: altruism, social-welfare concerns, self-interest, egalitarianism, spitefulness and inequality-seeking. For instance, all the (α, β) categories that include the value of 0 for both parameters are consistent with self-interest. The four cells representing these categories are surrounded by a green line. Also, the two (α, β) categories that include negative values of α and positive values of β are consistent with altruistic motives and are surrounded by a light blue line. As was suggested by the previous analyses, above-median CRTs are concentrated (55% of them) in the category “α ≤ 0, β ϵ [0, 0.5],” which is highlighted in Figure 2. The proportion of above-median CRT subjects belonging to this category is significantly larger than the proportion of above-median CRT subjects belonging to any other category (two-sided Normal Proportion tests, *p*s < 0.01). In the case of below-median CRT subjects a much lower proportion (29%) belong to the “α ≤ 0, β *ϵ* [0, 0.5]” category (two-sided Normal Proportion test, *p* < 0.01). This category is still the most populated category among below-median CRT individuals and the proportion of individuals belonging to this category is significantly larger than the proportion of below-median CRT individuals belonging to any other category (two-sided Normal Proportion tests, *p*s < 0.01) but the “α ≥ 0.5, β *ϵ* [0, 0.5]” category (*p* = 0.38).

The category “α ≤ 0, β ϵ [0, 0.5]” is consistent with both self-interest and altruism and can thus be seen as “weak altruism”. Our choice of terminology is to refer as “weak” all the social preferences categories that are consistent with self-interest (i.e., α = 0 and β = 0). We refer to as “strong” all the (α, β) social preferences categories which are not “weak.” Note that the “weak altruism” category is also the only category that is consistent with social-welfare motives. In order to show that these subjects display a preference for social welfare, however, one must show that −0.5 < α < 0 which cannot be demonstrated given the social preferences elicitation task used in this study.

**Figure 3:**
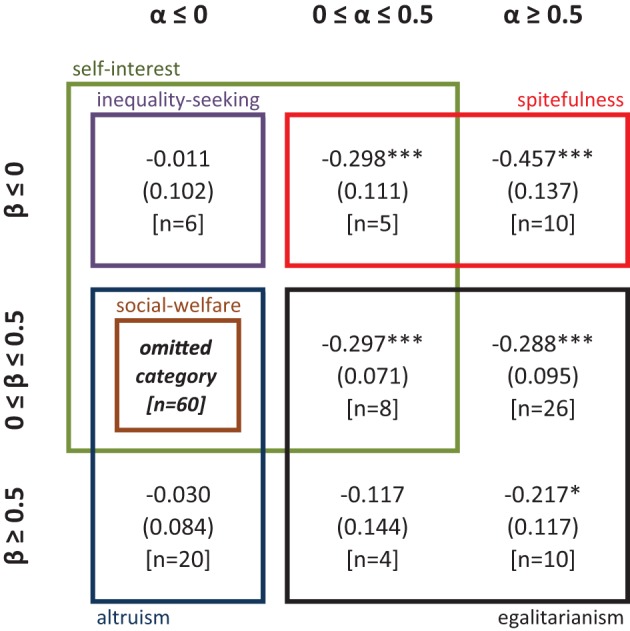
**Output of multinomial Probit regression (Study 1)**. In each cell, the figure shows the coefficient of CRT obtained by comparing that specific social preference category with “α ≤ 0, β ϵ [0, 0.5]”. ^*^, ^**^, ^***^ denote p-values lower than 0.10, 0.05, and 0.01, respectively. Ll = −247.017, Wald χ^2^ = 45.12 (*p* < 0.01), *N* = 149. Robust standard errors clustered on individuals are shown in parentheses, and the number of observations in square brackets.

In order to inquire further on the categorization of social preferences and highlight differences across CRT scores, we perform a multinomial Probit regression (see Figure 3). We estimate the likelihood that an individual is included in the category “α ≤ 0, β ϵ [0, 0.5]” as compared to each of the other eight categories. We include CRT scores and gender as regressors. In each cell representing an (α, β) category in Figure 3, we show the coefficient associated to CRT scores for the comparison of this specific (α, β) category with the omitted category (“α ≤ 0, β ϵ [0, 0.5]”). As expected, all the coefficients associated to CRT are negative, indicating that subjects with higher CRT scores are more likely to be included in the default category than in any of the other categories. These coefficients are significant (ps < 0.01) when comparing the default category with the following ones: “α ϵ [0, 0.5], β ≤ 0” (weakly spiteful), “α ϵ [0, 0.5], β ϵ [0, 0.5]” (weakly egalitarian) and “α ≥ 0.5, β ≤ 0” (strongly spiteful). The coefficients are close to significance when comparing the default option with the category “α ≥ 0.5, β ≥ 0.5” (strongly egalitarian, *p* = 0.06). However, CRT scores are not statistically significant when comparing the default category with the remaining three categories (ps > 0.41): “α ≤ 0, β ≥ 0.5” (strongly altruistic), “α ≤ 0, β ≤ 0” (weakly inequality seeking) and “α ϵ [0, 0.5], β ≥ 0.5” (strongly egalitarian). Yet, the latter two categories contain only six and four observations, respectively. Finally, only the coefficients of these three categories (−0.030, −0.011, and −0.117, respectively) are significantly (or close to significance) different from that of the strongly spiteful category “α ≥ 0.5, β ≤ 0” (*p* < 0.01, *p* < 0.01, and *p* = 0.06), which reports the highest coefficient in absolute value (−0.457).

Our classification thus suggests that high cognitive reflection is characteristic of individuals with α ≤ 0 and β ϵ [0, 0.5], which corresponds to “weak” altruism, whereas less reflective individuals are more likely to be guided by either spiteful or egalitarian motives. Yet, our previous analysis of each of the four decisions in the social preferences elicitation task led to the more precise conclusion that high CRTs are characterized by α < 0 and β ϵ (0, 0.5). That is, high-CRT individuals are unlikely to be purely selfish (α = 0, β = 0); instead they can be considered as *mildly altruistic*.

Given the data of Study 1, high-CRT people are apparently more willing to give money to the other person than low-CRT people as long as it is not too costly for them to do so. Indeed, subjects with higher CRT scores are more willing to give money to the other person when it is costless (Decisions 1, 3, and 4) but not when it is very costly (Decision 2). Moreover, note that those subjects who give money to the other person in Decisions 1, 3, and 4 may respond to concerns for social welfare whereas such interpretation of giving is not valid for Decision 2.

However, substantial differences may still exist in the levels of envy (α) and compassion (β) among those subjects characterized as mildly altruistic. Some mildly altruistic individuals may be close to selfishness (α ≈ 0, β ≈ 0) whereas others may not. Our data cannot separate these different types of subjects. To that end, we extend the social preferences elicitation task of Bartling et al. (2009) in our second study. First, we include in our elicitation task a decision for which increasing the payoff of the other person above one's own is personally costly. This decision will allow us to isolate subjects who are practically selfish (α ≈ 0) in the negative domain of envy. Second, among mildly altruistic subjects there may be individuals with social-welfare concerns (|α_*i*_|, |β_*i*_| < 0.5). To isolate people who care about social welfare, we need that increasing the *better-off* counterpart's payoff in the aforementioned decision also increases social welfare (i.e., the cost for the decision maker is lower than the increase in the other player's payoff). In addition, we need to include another decision for which increasing a *worse-off* counterpart's payoff at a personal cost also increases social welfare.

In order to dig into these issues and obtain a more refined assessment of the values of α and β, we thus modified the social preferences task of Bartling et al. (2009) by adding two decisions which were designed along the lines of the previous discussion. This modified task was implemented in Study 2.

## Study 2

### Methods

#### Participants and general protocol

Participants were 158 students [51.90% female; mean age 21.52 ± 2.63 (SD)] from the University Carlos III of Madrid in Spain. Participants were enrolled in the following majors at the time of the study: Business and Economics (51.9%), Law School (28.5%), Humanities and Social Sciences (5.7%), Science and Technology (11.4%), and other studies (2.5%). These participants were recruited from a database of more than 2500 students. We conducted a total of eight sessions, three with 18 and 20 participants each and two with 22 participants. On average, sessions lasted for 60 min. As in Study 1, all subjects completed the same tasks in the same order and the social preferences elicitation task was performed before the CRT. In between the social preferences task and the CRT, participants completed a series of unrelated tasks for about 15 min and had a break of 10 min (see Text S1). All participants in the experiments reported in this Study agreed to the Participation Rules and Privacy Policy when they registered to participate in experiments. Anonymity was always preserved (in agreement with Spanish Law 15/1999 on Personal Data Protection) by randomly assigning a numerical code to identify the participants in the system. No association was ever made between their real names and the results. As is standard in socio-economic experiments, no ethic concerns are involved other than preserving the anonymity of participants. No deception was used. This procedure was checked and approved by the department of Economics of the University Carlos III of Madrid; the institution hosting the experiments. At that time no official IRB was established at the university.

#### Cognitive style assessment

As in Study 1, participants completed the extended version of the CRT developed by Toplak et al. (2014). In Table S2, we display the % of subjects answering each question correctly, split by gender. Again, males scored higher on the test than females and these differences were statistically significant (see Table S2). The full distribution of correct answers by males (mean = 3.22 ± 1.73) and females (mean = 2.18 ± 1.35) is provided in Figure S2. The test was not incentivized.

#### Social preferences elicitation

Participants made six choices between two possible allocations of money between themselves and another anonymous participant with whom they were randomly matched. Similarly to Study 1, in each experimental session, two participants and one of the six decisions were selected at random for payment. The choice of one of the two participants in the selected decision was used to allocate payoffs between the two participants. All decisions were anonymous. The first four decisions used the exact same payoffs as in Bartling et al. (2009). Decisions 5 (advantageous domain) and 6 (disadvantageous domain) were designed for this particular experiment in such a way that the decision maker could increase the payoff of the other participant by €6 at a €2 cost. Thus, the cost for the decision-maker is low relative to the increase of the other's payoff (i.e., a 1:3 cost-to-benefit ratio) so that giving also increases social welfare. The new task allows us to disentangle four subcategories of the “weak altruism” category of Study 1 (α ≤ 0, β ϵ [0, 0.5]) which was the most populated category and also the only one which was consistent with welfare concerns. In Study 2 and in contrast to Study 1, we could identify subjects exhibiting combinations of α and β that are consistent with social-welfare concerns but *not* with self-interest. We could thus distinguish between “weak” and “strong” preferences for social welfare.

In particular, the 1:3 cost-to-benefit ratio used in Decision 5 allows us to break down the β ϵ [0, 0.5] category into two subcategories ([0, 0.25] and [0.25, 0.5]) thus refining our estimation of individual social preferences. Note that the payoffs used in Study 1 had to be increased in order to break down the β ϵ [0, 0.5] category while also avoiding negative or non-integer payoffs. To accomplish this, we decided to use the original payoffs of Bartling et al. (2009). Given that utility is assumed to be linear over the relevant range of payoffs (Fehr and Schmidt, 1999), it is important to point out that it is not the absolute but the relative change in payoffs which determines the values of the model parameters in each case. For the sake of symmetry, we used the same 1:3 cost-to-benefit ratio for Decision 6 so that we could estimate values of α below as well as above −0.25. All the allocation decisions are described in Table 3. Option A always yielded an even distribution of money (€10 to both the self and the other participant) whereas option B yielded uneven payoffs. For each decision, we show in parentheses the envy/compassion parameter associated to choosing the egalitarian and non-egalitarian options (i.e., options A and B) and in square brackets the proportion of subjects who chose each option. Note that the model parameters associated to Decisions 1–4 are the same as in Study 1, except for the fact that in Decision 4 the threshold for the envy parameter is now 0.125 instead of 0.5. However, given that the categorization of social preference types does not depend on the exact value of α (provided that it is positive), whether 0.125 or 0.5 is used as threshold should not interfere with the goal of our study.

**Table 3:** **Decisions in the social preferences task (Study 2)**.

**Decision #**	**Option A self** **other**		**Option B self** **other**	
1	€10,€10	(β ≥ 0) [86%]	€10,€6	(β ≤ 0) [14%]
2	€10,€10	(β ≥ 0.5) [27%]	€16,€4	(β ≤ 0.5) [73%]
3	€10,€10	(α ≥ 0) [42%]	€10,€18	(α ≤ 0) [58%]
4	€10,€10	(α ≥ 0.125) [30%]	€11,€19	(α ≤ 0.125) [70%]
5	€10,€10	(β ≥ 0.25) [42%]	€12,€4	(β ≤ 0.25) [58%]
6	€10,€10	(α ≥ −0.25) [84%]	€8,€16	(α ≤ −0.25) [16%]

For each option, we display the payoff for the decision-maker and the recipient, the associated model parameters (in parentheses) and the % of subjects choosing it (in square brackets). N = 158.

## Results and discussion

### Decision analysis

**Figure 4:**
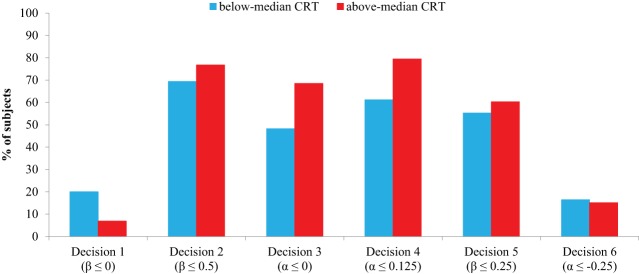
**Percentage of subjects choosing option B in each decision, by CRT groups (Study 2)**. The model parameters associated to option B are shown in parentheses.

Decisions 1–4 as reported in Table 3 mimic very closely the results of Study 1 (see Table 1) suggesting that our original results are remarkably robust to eliciting social preferences in a different country with a different set of payoffs. Table 4 reports the results of a series of Probit regressions where the choice of option B in each decision is regressed as a function of CRT scores, controlling for gender. Robust standard errors clustered on individuals are presented in parentheses, and marginal effects in square brackets. As in Study 1, the results are fairly similar if we account for (correlated) multiple comparisons using multivariate Probit (see Tables S4, S6). In Figure 4, we display the proportion of subjects choosing option B in each decision, for individuals with below-median (i.e., two or less correct answers, *n* = 85) and above-median (*n* = 73) CRT scores.

**Table 4:** **Non-egalitarian choice (option B) as a function of CRT (Study 2)**.

	**Decision 1**	**Decision 2**	**Decision 3**	**Decision 4**	**Decision 5**	**Decision 6**
**Dep var:**	**β ≤ 0**	**β ≤ 0.5**	**α ≤ 0**	**α ≤ 0.125**	**β ≤ 0.25**	**α ≤ −0.25**
	**(vs ≥ 0)**	**(vs ≥ 0.5)**	**(vs ≥ 0)**	**(vs ≥ 0.125)**	**(vs ≥ 0.25)**	**(vs ≥ −0.25)**
	**(1)**	**(2)**	**(3)**	**(4)**	**(5)**	**(6)**
CRT	−0.149^*^	0.122^*^	0.124^*^	0.150^**^	0.109	−0.044
	(0.088)	(0.073)	(0.069)	(0.072)	(0.067)	(0.084)
	[−0.031^*^]	[0.040^*^]	[0.047^*^]	[0.049^**^]	[0.042^*^]	[−0.011]
Female	0.306	0.070	−0.285	−0.462^**^	0.294	−0.146
	(0.276)	(0.227)	(0.213)	(0.223)	(0.216)	(0.257)
	[0.063]	[0.023]	[−0.108]	[−0.151^**^]	[0.113]	[−0.035]
Cons	−0.898^***^	0.252	0.017	0.390	−0.250	−0.812^***^
	(0.334)	(0.271)	(0.262)	(0.264)	(0.260)	(0.315)
	[0.184]	[0.599]	[0.507]	[0.652]	[0.401]	[0.208]
ll	−60.929	−90.969	−103.938	−90.902	−105.927	−68.750
Wald χ^2^	6.18^**^	2.89	6.86^**^	10.74^***^	3.35	0.44
Pseudo R^2^	0.045	0.017	0.035	0.063	0.016	0.004
N	158	158	158	158	158	158

Probit estimates. The α and β parameters associated with the dependent variable are displayed on top of each column. Robust standard errors clustered on individuals are shown in parentheses and average marginal effects of the explanatory variables are shown in square brackets (for the constant, this value represents the probability obtained from normal transformation of the Probit coefficient).

*, **, *** denote p-values lower than 0.10, 0.05, and 0.01, respectively.

We find that CRT is negatively related to choosing option B in Decision 1, indicating that individuals with higher CRT scores are less likely to display β ≤ 0, in line with the findings in Study 1. Although this relationship is only close to significance (*p* = 0.09), it is worth noticing that the marginal effect of CRT is 3.1% and the mean proportion of non-egalitarian choice in Decision 1 is 14%, which results in a strong size effect remarkably similar to that obtained in Study 1 (i.e., 2.9%). Moreover, the effect of cognitive reflection on Decision 1 is statistically significant (*p* = 0.04, Table S8, column 1) when the binary categorization is used as explanatory variable. As in Study 1, a two-sided binomial test rejects the hypothesis that individuals are indifferent between the two options (for both below- and above-median CRT scores, the proportion of subjects choosing option B is well below 50%; *p*s < 0.01; see Figure 4). That is, the majority of subjects, especially those with higher CRT scores, seem to display β>0.

In Decision 2, we observe some discrepancy with respect to Study 1 where the effect of CRT was negative although not significant. In Study 2, CRT scores are positively related to choosing option B, indicating that higher CRT individuals are more likely to exhibit β ≤ 0.5. Yet, this relationship is only close to significance (*p* = 0.09) and even turns insignificant when the binary categorization of CRT is used (*p* = 0.32, Table S8, column 2). As in Study 1, the proportion of subjects choosing option B in Decision 2 is higher than 50% in both CRT groups (two-sided binomial tests, *p*s < 0.01; see Figure 4). That is, the majority of subjects, especially those with higher CRT scores, seem to be characterized by β < 0.5.

With regards to Decisions 3 and 4, the results are similar to those of Study 1. Specifically, CRT is positively associated with the choice of option B in Decision 3, implying α ≤ 0. Although this relationship falls short of significance in Table 4 (*p* = 0.07), it turns significant when the binary CRT variable is used (*p* = 0.03, Table S8, column 3). From Figure 4, we see that roughly 48% of below-median CRT subjects choose option B in Decision 3 (which is not significantly different from 50%, two-sided binomial test, *p* = 0.66, so we cannot reject that they are, on average, indifferent between both options: α = 0). In contrast, 68% of above-median CRT subjects choose option B (which is significantly different from 50%, *p* < 0.01). So, high-CRT subjects seem to display α < 0. In Decision 4, the choice of option B is positively and significantly predicted by CRT (*p* = 0.04; the binary CRT categorization yields *p* = 0.05, Table S8, column 4), implying that higher CRT subjects are more likely to display α ≤ 0.125. Indeed, about 79% of above-median CRT subjects choose option B in Decision 4 (Figure 4), which is significantly different from 50% (two-sided binomial test, *p* < 0.01), whereas 61% of below-median CRT subjects did so (which is also significantly different from 50%, *p* = 0.05). Thus, α < 0.125 seems to best characterize the majority of subjects, especially those with high CRT scores.

In Decision 5, CRT does not yield a significant effect (*p* = 0.11; using the binary CRT variable, *p* = 0.39, Table S8, column 5). While 60% of above-median CRT subjects choose option B in Decision 5 (this proportion is close to be significantly different from 50%, two-sided binomial test, *p* = 0.10), this percentage shrinks to 55% for below-median CRT subjects (not significantly different from 50%, *p* = 0.38) (Figure 4). This indicates that most high-CRT subjects are characterized by β < 0.25, whereas the median β seems to be close to 0.25 for low-CRT subjects. Finally, in Decision 6, where option B implies α ≤ −0.25, the coefficient associated to CRT is far from significant (*p* = 0.60; also using the binary CRT variable, *p* = 0.74, Table S8, column 6). About 16% of below-median CRT subjects and 15% of above-median CRT subjects choose option B in Decision 6 (both proportions are significantly different from 50%, two-sided binomial tests, *p*s < 0.01; see Figure 4), which implies that the majority of subjects is best characterized by α>−0.25, regardless of CRT scores.

In sum, the previous analysis suggests that high-CRT individuals are best described by α ϵ (−0.25, 0) and β ϵ (0, 0.25), whereas the distribution of the envy and compassion parameters of low CRT subjects is much more disperse.

### Social preferences categorization

**Figure 5:**
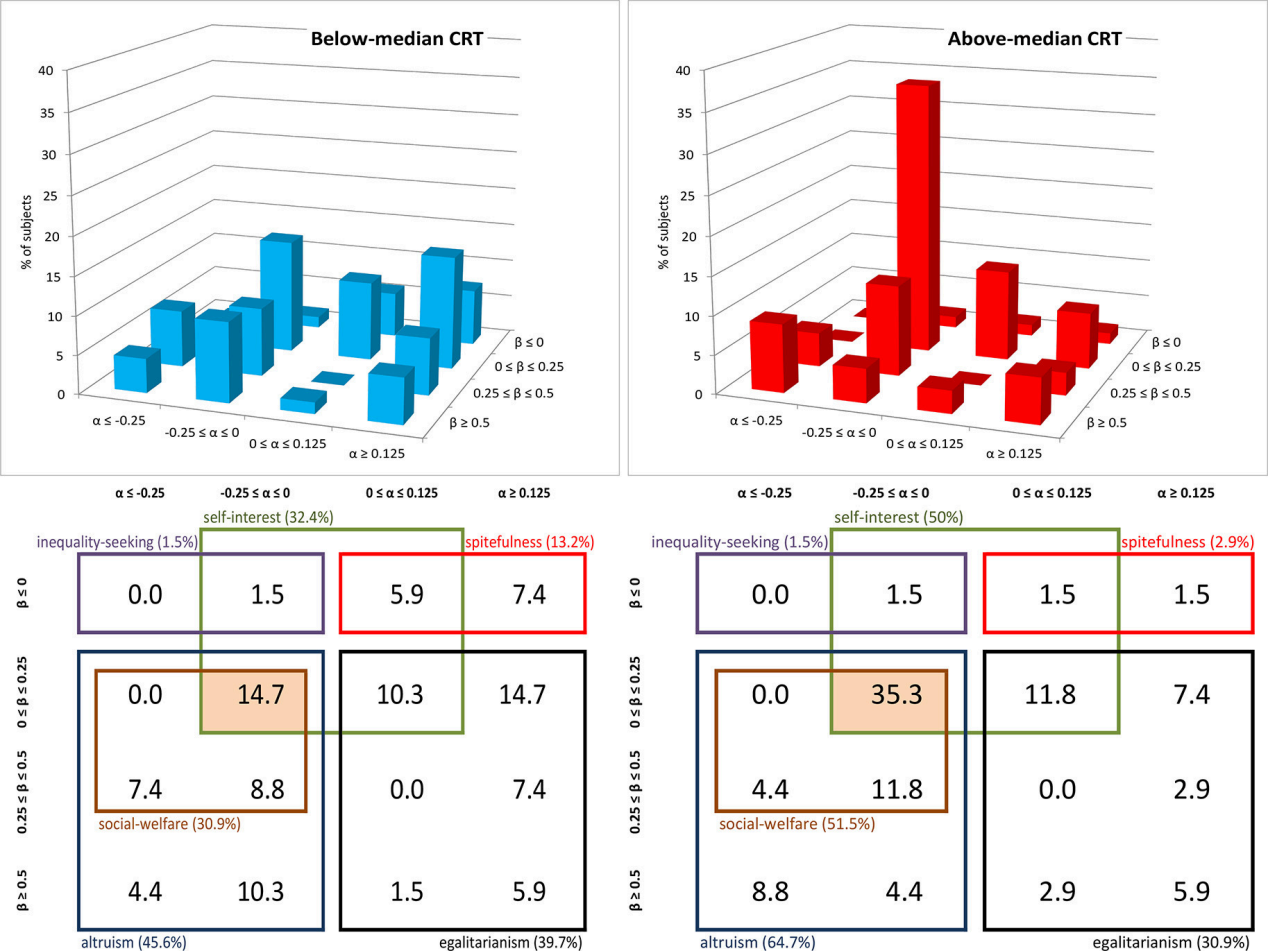
**Classification of subjects according to the envy and compassion parameters, by CRT groups (Study 2)**. The figure displays the % of subjects that can be classified according to each combination of α and β and the social preferences which are consistent with each category, broken down into below-median (*n* = 68) and above-median (*n* = 68) CRT score groups.

Now, we proceed by categorizing each individual according to their social preferences. In Figure 5, we display the proportion of subjects that are characterized by each of the 16 combinations of the envy and compassion parameters. We represent below-median CRT subjects on the left panel and above-median CRT subjects on the right panel. In our social preferences categorization, we excluded 22 subjects whose choices were inconsistent, so we ended up with 136 observations (68 below-median and 68 above-median CRT subjects). No individuals were assigned to the following categories: “α ≤ −0.25, β ≤ 0” (strongly inequality seeking), “α ≤ −0.25, β ϵ [0, 0.25]” (strongly altruistic with social-welfare concerns) and “α ϵ [0, 0.125], β ϵ [0.25, 0.5]” (strongly egalitarian).

As expected, above-median CRT subjects are concentrated (35% of them) in the category “α ϵ [−0.25, 0], β ϵ [0, 0.25],” which again represents “weak altruism,” whereas below-median CRT subjects are more dispersed across categories, similarly to Study 1. The proportion of above-median CRT subjects belonging to this category is significantly larger than the proportion of above-median CRT subjects belonging to any other category (two-sided Normal Proportion test, *p*s < 0.01). In the case of below-median CRT subjects a much lower proportion of people (15%) belong to the “α ≤ 0, β ϵ [0, 0.5]” category (*p* < 0.01). This category is still the most populated category among below-median CRT subjects but the proportion of below-median CRT subjects belonging to this category is only significantly larger than six out of the fifteen other categories. Note that, in contrast to Study 1 where there was only one category consistent with social-welfare concerns, Study 2 allows us to identify different degrees of such concerns. The category defining the majority of above-median CRT subjects (“α ϵ [−0.25, 0], β ϵ [0, 0.25]”) corresponds to “weak” social-welfare concerns.

**Figure 6:**
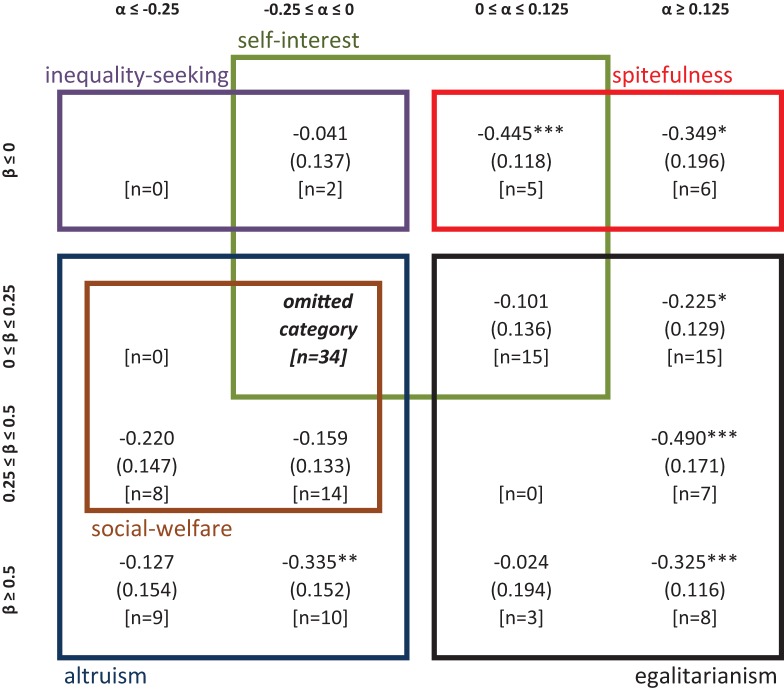
**Output of multinomial Probit regression (Study 2)**. In each cell, the figure shows the coefficient of CRT obtained by comparing that specific social preference category with “α ϵ [−0.25, 0], β ϵ [0, 0.25]”. ^*^, ^**^, ^***^ *p*-values lower than 0.10, 0.05, and 0.01, respectively. Ll = −300.325, Wald χ^2^ = 50.17 (*p* < 0.01), *N* = 136. Robust standard errors clustered on individuals are shown in parentheses, and the number of observations in square brackets.

In order to further explore these observations, we conducted a multinomial Probit regression, the results of which are presented in Figure 6. As for Study 1, CRT and gender are used as regressors. The most populated category, “α ϵ [−0.25, 0], β ϵ [0, 0.25],” is used as the default category for the regression analysis. The numbers inside the remaining cells indicate the effect of CRT score on the likelihood that an individual is included in this specific category as compared to the default category. As expected, all estimates are negative indicating that subjects with higher CRT scores are more likely to belong to the default category “α ϵ [−0.25, 0], β ϵ [0, 0.25]” than to the remaining categories. The effect of CRT is statistically significant when comparing the default category to the following ones: “α ϵ [−0.25, 0], β ≥ 0.5” (strongly altruistic, *p* = 0.02), “α ϵ [0, 0.125], β ≥ 0” (weakly spiteful, *p* < 0.01), “α ≥ 0.125, β ϵ [0.25, 0.5]” (strongly egalitarian, *p* < 0.01) and “α ≥ 0.125, β ≥ 0.5” (strongly egalitarian, *p* < 0.01). The effect of CRT is close to significance with respect to “α ≥ 0.125, β ≥ 0” (strongly spiteful, *p* = 0.08) and “α ≥ 0.125, β ϵ [0, 0.25]” (strongly egalitarian, *p* = 0.08) and with respect to “α ≤ −0.25, β ϵ [0.25, 0.5]” (strongly altruistic with social-welfare concerns, *p* = 0.13). The five remaining categories did not yield significant CRT effects (ps > 0.23). An interesting difference of Study 2 with respect to Study 1 is that two of the “strong altruism” categories show significant (or nearly significant) differences with the default group. This did not happen in Study 1, where there was only one such category (namely “α ≤ 0, β ≥ 0.5”) for which the associated coefficient was largely insignificant. Note that here the strongest difference is given by the comparison with the following category “α ≥ 0.125, β ϵ [0.25, 0.5]” (strongly egalitarian), which is the category higher CRT subjects are less likely to belong to. However, the coefficient associated to this category only differs significantly from the coefficient of the following categories: “α ϵ [−0.25, 0], β ≤ 0” (weakly inequality seeking; note that only two subjects belong to this category), “α ϵ [0, 0.125], β ϵ [0, 0.25]” (weakly egalitarian), and “α ϵ [0, 0.125], β ≥ 0.5” (strongly egalitarian; only three subjects belong to this category) (ps < 0.05). The coefficient associated to the “α ≥ 0.125, β ϵ [0.25, 0.5]” category also differs from the coefficients of the following categories: “α ≤ −0.25, β ≥ 0.5” (strongly altruistic) and “α ϵ [−0.25, 0], β ϵ [0.25, 0.5]” (strongly altruistic with social-welfare concerns), although these differences are only close to significance (ps = 0.08).

Taken together, the results of Study 2 indicate that high cognitive reflection is characteristic of individuals who make choices consistent with mildly altruistic motives that increase social welfare at a *very low* cost. Low cognitive reflection is characteristic of individuals who make decisions consistent with either egalitarian or spiteful motives. These findings are consistent with Study 1. In slight contrast to Study 1, however, low-CRT people are also associated with strong altruistic motivations. It is important to note that, in Study 2, we were able to split the weak altruism/social-welfare preferences category into four subcategories. In contrast to Study 1, we could therefore isolate strong social-welfare concerns from weak social-welfare concerns and conclude that it is the latter which best characterizes individuals with high CRT scores. This methodological feature of Study 2 may thus have facilitated the observation of a difference in terms of CRT scores between those subjects included in the default category and those classified as strongly altruistic.

## General discussion

### Cognitive reflection and social preferences: Our insights

In two studies, we showed that those individuals with a more reflective cognitive style (i.e., those who are less likely to rely on intuitive, System 1 processes) are more likely to make choices consistent with mildly altruistic motives in simple monetary decisions free of strategic and reciprocal concerns. These results suggest that behaviors that increase social welfare by increasing others' payoffs at a very low or no cost for the individual may be the result of conscious deliberation rather than automatic heuristics. Behaviors driven by egalitarian or spiteful concerns, however, appear to be more intimately associated with intuition^6^.

While the above findings are robust across the two studies, we also find a slight but remarkable difference with respect to strongly altruistic choices that increase the other's payoff at a relatively high cost to the individual. In Study 1 reflective subjects were quite likely to make such choices whereas in Study 2 they were not. This may be partly explained by differences in stakes across studies, although our estimation procedure relies on the assumption that utility is linear over the relevant range of payoffs (as in Fehr and Schmidt, 1999) in which case stakes would not affect social preferences decisions. Methodological differences across studies (in Study 2 weak altruism was divided into four subcategories and strong altruism into two subcategories) may also have facilitated the observation of this divergence. In addition, this difference might also be accounted for by either students' educational backgrounds (majors) or cultural differences (Study 1 was conducted in the US while Study 2 was conducted in Spain). Interestingly, no differences in giving behavior between US and Spain student subjects were documented in the baseline experiments conducted by Rey-Biel et al. (2015) suggesting that cultural differences in giving may not play a major role in our findings^7^. Finally, this difference could also be explained by the existence of ceiling effects as the average level of cognitive reflection, as measured by the number of correct answers to the CRT, was higher (25% higher, two-sided *t*-test: *p* < 0.01) in Study 1. Exploring these possibilities is an interesting avenue for future research.

### Toward reconciliation: A unified view of the cognitive basis of social behavior

At first sight, it might seem that more reflective individuals are guided by “weaker” social motivations as they are typically less likely to be classified in the categories representing strong social preferences. Accordingly, it may be tempting to interpret our findings as evidence that cognitive reflection goes along with self-interest in (non-strategic) one-shot social interactions. This would be, however, an incorrect interpretation of our findings because self-interest cannot explain why the most reflective individuals are overwhelmingly characterized as “mildly” altruistic while not being affected by other social preferences like spitefulness or egalitarianism. Therefore, it is not self-interest *per se* but a very particular mixture of self-interest and altruistic/social-welfare concerns that characterizes reflective individuals. In terms of the parameters of the generalized version of the Fehr–Schmidt's model (Fehr and Schmidt, 1999) used here, high cognitive reflection is associated with a combination of slightly negative values of envy (α) and slightly positive values of compassion (β). Similar results have been obtained through structural estimation of the individuals' envy and compassion parameters in Ponti and Rodriguez-Lara (2015). Moreover, there are much less individual differences in these parameters among individuals with high CRT scores than among individuals with low CRT scores. While mean values of envy appear to be higher for individuals with a less reflective cognitive style, the relationship between CRT scores and compassion is more complex. Indeed, either high or very low (even negative) values of β can be associated with low cognitive reflection. Thus, we would not have been able to uncover some of the key differences between groups if we had focused on estimating mean values of the model parameters.

From the viewpoint of the Social Heuristics Hypothesis (Rand et al., 2014), our results suggest that behaviors driven by either egalitarianism or spitefulness (and possibly strong altruism) may be internalized as heuristics, which ultimately implies that they may be, on average, advantageous in daily-life interactions. Indeed, neurobiological research indicates that humans experience psychological satisfaction from observing equitable outcomes (Tricomi et al., 2010; Zaki and Mitchell, 2011) but also from out-earning others (Fliessbach et al., 2007; Bault et al., 2011), even if their own absolute payoff is unaffected. On the other hand, reflection should lead people to adapt their decision rules to the environment at hand (e.g., Kahneman, 2011). Under this logic, the present results indicate that the most adaptive decisions in one-shot, non-strategic social interactions are those guided by mildly altruistic motives.

These findings can shed light on the current debate regarding whether (pro)social behavior is automatic or deliberate (Rand and Nowak, 2013; Zaki and Mitchell, 2013). Previous research has led to ostensibly contradictory results which have partly been accounted for by the existence of moderator variables (e.g., subjects' prior experience in economic experiments; Cone and Rand, 2014; Rand et al., 2014, 2015) and confounding factors (linked, for example, to the use of reaction times to infer the effect of reflection on behavior; Recalde et al., 2014; Evans et al., 2015; Krajbich et al., 2015). Yet, our findings reveal that another non-negligible portion of these apparently conflicting findings can be reconciled by accounting for two often-ignored factors. First, different motives can lead to identical choices in the experimental set-ups normally used to infer the nature of social behavior (Charness and Rabin, 2002). Second, by putting the focus almost exclusively on the conflict between prosociality and self-interest, previous research has tended to overlook antisocial motivations that can trigger behaviors which may appear as selfish or even prosocial (e.g., Espín et al., 2012; Brañas-Garza et al., 2014).

In sum, our findings highlight that the analysis of the cognitive basis of social behavior is likely to be more complex than previously thought. It must also be said, however, that strategic issues and reciprocity (which were voluntarily absent of our study) may play an essential role in social dilemma and ultimatum games (e.g., Charness and Rabin, 2002; Falk and Fischbacher, 2006; Fehr and Schmidt, 2006), blurring further the analysis of the cognitive basis of social behavior (Rand and Nowak, 2013).

Finally, our results are based on a trait approach to cognitive reflection, which by definition has to be addressed in a correlational manner thus leaving open concerns about causality and about the existence of third-variable confounds. It would therefore be important for future research to assess the robustness of these findings to experimental manipulations of intuitive processing. Identifying the neurobiological underpinnings of these individual differences in trait reflectiveness and their relationship to social preferences appears as a necessary next step toward achieving a more complete understanding of the cognitive basis of human social behavior (Nash et al., 2015).

### Conflict of interest statement

The authors declare that the research was conducted in the absence of any commercial or financial relationships that could be construed as a potential conflict of interest.

## Footnotes

^1^This may be the case, e.g., when there exist high levels of environmental uncertainty/volatility (e.g., violence) or when local (vs. global) competition for resources prevails (Gardner and West, 2004; McCullough et al., 2012; Sylwester et al., 2013; Prediger et al., 2014).

^2^Note that we are using preference types as a classification device, irrespective of whether or not these are completely stable characteristics of individuals as often assumed in the social preferences literature. Indeed a number of studies challenge such interpretation and suggest that social preferences can be modulated/manipulated (e.g., Rand et al., 2012; McCall et al., 2014).

^3^Also, the CRT has been found to correlate with one's ability to delay gratification (Frederick, 2005; Bosch-Domènech et al., 2014) and avoid distractions at work (Corgnet et al., in press). In addition, student performance in the CRT has been shown to correlate positively with earnings in experimental asset markets (Corgnet et al., 2015) and other individual tasks involving the capacity to think backwards (Brañas-Garza et al., 2012a). Finally, the CRT has also been shown to correlate negatively with the adoption of paranormal beliefs (Pennycook et al., 2012; Shenhav et al., 2012).

^4^Note that an individual with α ≤ −0.5 and β ≥ 0.5 would give money away even when doing so does not increase the total surplus (i.e., social welfare, also referred to as “efficiency”).

^5^Variance inflation factors above 10 are typically associated with collinearity issues (e.g., Neter et al., 1989; Kennedy, 2008; Hair et al., 2009). The lowest threshold for the variance inflation factor we are aware of is 4 (see O'Brien, 2007). Even this lower threshold would not be met in our case.

^6^In a related strand of research on the psychological underpinnings of social behavior, Espín et al. (2012, 2015) have shown that those individuals who discount the future more heavily display more spite-based but *not* egalitarian behavior in economic games (namely in a dual-role ultimatum game and a social dilemma game with punishment). Since we find that lower CRT scores are related to both spitefulness and egalitarianism, our results thus support Espín et al.'s arguments that the social behaviors associated with short-run (vs. long-run) goals differ from those associated with intuitive (vs. controlled) processes (even though temporal discounting is negatively correlated with CRT scores; see Frederick, 2005; Bosch-Domènech et al., 2014).

^7^The authors found differences across samples only in the case in which the determinants (work or luck) of participants' income (the endowment of participants in our case) were not known.
