# Supplementary material for: The cognitive basis of social behavior: cognitive reflection overrides antisocial but not always prosocial motives
Source: Front Behav Neurosci. 2015 Nov 5;9:287. doi: 10.3389/fnbeh.2015.00287 (PMC4633515; doi:10.3389/fnbeh.2015.00287)
Supplement: Supplementary file 1 [file TableS1.DOCX]

| CRT question | Males (%) | Females (%) | p-value |
| --- | --- | --- | --- |
| 1 | 56.63 | 34.33 | <0.01 |
| 2 | 44.58 | 28.36 | 0.04 |
| 3 | 60.24 | 35.82 | <0.01 |
| 4 | 49.40 | 32.84 | 0.05 |
| 5 | 42.17 | 19.40 | <0.01 |
| 6 | 48.19 | 38.81 | 0.32 |
| 7 | 66.27 | 49.25 | 0.04 |

**Table S1. Percentage of subjects answering correctly the CRT by question and gender (Study 1).** P-values from two-sided Fisher’s exact tests for the (gender) difference in proportions.
