# Supplementary material for: The cognitive basis of social behavior: cognitive reflection overrides antisocial but not always prosocial motives
Source: Front Behav Neurosci. 2015 Nov 5;9:287. doi: 10.3389/fnbeh.2015.00287 (PMC4633515; doi:10.3389/fnbeh.2015.00287)
Supplement: Supplementary file 2 [file TableS2.DOCX]

| CRT question | Males (%) | Females (%) | p-value |
| --- | --- | --- | --- |
| 1 | 47.37 | 31.71 | 0.05 |
| 2 | 38.16 | 18.29 | <0.01 |
| 3 | 43.42 | 17.07 | <0.01 |
| 4 | 47.37 | 24.39 | <0.01 |
| 5 | 42.11 | 25.61 | 0.03 |
| 6 | 72.37 | 47.56 | <0.01 |
| 7 | 31.58 | 53.66 | <0.01 |

**Table S2. Percentage of subjects answering correctly the CRT by question and gender (Study 2).** P-values from two-sided Fisher’s exact tests for the (gender) difference in proportions.
