# Supplementary material for: The cognitive basis of social behavior: cognitive reflection overrides antisocial but not always prosocial motives
Source: Front Behav Neurosci. 2015 Nov 5;9:287. doi: 10.3389/fnbeh.2015.00287 (PMC4633515; doi:10.3389/fnbeh.2015.00287)
Supplement: Supplementary file 3 [file TableS3.DOCX]

| *N = 150* | Decision 2 | Decision 3 | Decision 4 |
| --- | --- | --- | --- |
| Decision 1 | 0.218*** | -0.241*** | -0.142* |
| Decision 2 | - | -0.009 | -0.022 |
| Decision 3 |  | - | 0.765*** |

**Table S3. Correlation coefficients (Pearson) for all decisions in the social preferences elicitation task (Study 1).** *, **, *** denote p-values lower than 0.10, 0.05 and 0.01, respectively.
