# Supplementary material for: The cognitive basis of social behavior: cognitive reflection overrides antisocial but not always prosocial motives
Source: Front Behav Neurosci. 2015 Nov 5;9:287. doi: 10.3389/fnbeh.2015.00287 (PMC4633515; doi:10.3389/fnbeh.2015.00287)
Supplement: Supplementary file 4 [file TableS4.DOCX]

| *N = 158* | Decision 2 | Decision 3 | Decision 4 | Decision 5 | Decision 6 |
| --- | --- | --- | --- | --- | --- |
| Decision 1 | -0.001 | -0.210*** | -0.211*** | 0.123 | 0.026 |
| Decision 2 | - | -0.064 | -0.002 | 0.482*** | -0.241*** |
| Decision 3 |  | - | 0.519*** | -0.140* | 0.267*** |
| Decision 4 |  |  | - | -0.066 | 0.173** |
| Decision 5 |  |  |  | - | -0.400*** |

**Table S4. Correlation coefficients (Pearson) for all decisions in the social preferences elicitation task (Study 2).** *, **, *** denote p-values lower than 0.10, 0.05 and 0.01, respectively.
