# Supplementary material for: The cognitive basis of social behavior: cognitive reflection overrides antisocial but not always prosocial motives
Source: Front Behav Neurosci. 2015 Nov 5;9:287. doi: 10.3389/fnbeh.2015.00287 (PMC4633515; doi:10.3389/fnbeh.2015.00287)
Supplement: Supplementary file 5 [file TableS5.DOCX]

|  | Decision 1 | Decision 2 | Decision 3 | Decision 4 |
| --- | --- | --- | --- | --- |
| Dep var: | *β* ≤ 0 (vs ≥ 0) | *β* ≤ 0.5 (vs ≥ 0.5) | *α* ≤ 0 (vs ≥ 0) | *α* ≤ 0.5 (vs ≥ 0.5) |
|  | (1a) | (2a) | (3a) | (4a) |
| CRT | -0.125** | -0.062 | 0.255*** | 0.275*** |
|  | (0.062) | (0.052) | (0.058) | (0.062) |
| female | -0.541* | -0.268 | -0.217 | -0.217 |
|  | (0.298) | (0.240) | (0.226) | (0.241) |
| cons | -0.514* | 1.094*** | -0.452** | -0.452** |
|  | (0.269) | (0.251) | (0.229) | (0.229) |
| ll | -263.029 | | | |
| Wald χ^2^ | 30.43*** | | | |
|  | (1b) | (2b) | (3b) | (4b) |
| Raven | -0.057 | 0.028 | 0.101** | 0.138** |
|  | (0.060) | (0.055) | (0.050) | (0.053) |
| female | -0.413 | -0.187 | -0.478** | -0.451** |
|  | (0.269) | (0.232) | (0.212) | (0.225) |
| cons | -0.099 | 0.438 | -1.055 | -1.295 |
|  | (0.886) | (0.808) | (0.755) | (0.791) |
| ll | -274.920 | | | |
| Wald χ^2^ | 18.38** | | | |
|  | (1c) | (2c) | (3c) | (4c) |
| CRT | -0.122* | -0.100* | 0.256*** | 0.262*** |
|  | (0.070) | (0.056) | (0.063) | (0.066) |
| Raven | -0.008 | 0.077 | -0.003 | 0.040 |
|  | (0.070) | (0.059) | (0.053) | (0.060) |
| female | -0.538* | -0.302 | -0.217 | -0.223 |
|  | (0.299) | (0.239) | (0.228) | (0.242) |
| cons | -0.408 | 0.103 | -0.409 | -0.698 |
|  | (0.949) | (0.822) | (0.712) | (0.820) |
| ll | -261.771 | | | |
| Wald χ^2^ | 37.31*** | | | |
| N | 150 | | | |

**Table S5. Non-egalitarian choice (option B) as a function of CRT and Raven (Study 1).** Multivariate Probit estimates. The *α* and *β* parameters associated with the dependent variable are displayed on top of each column. In “a” regressions, the main explanatory variable is CRT score. In “b” regressions, the main explanatory variable is Raven score. In “c” regressions, both CRT and Raven scores are included as explanatory variables. Robust standard errors clustered on individuals are shown in parentheses. *, **, *** denote p-values lower than 0.10, 0.05 and 0.01, respectively.
