# Supplementary material for: The cognitive basis of social behavior: cognitive reflection overrides antisocial but not always prosocial motives
Source: Front Behav Neurosci. 2015 Nov 5;9:287. doi: 10.3389/fnbeh.2015.00287 (PMC4633515; doi:10.3389/fnbeh.2015.00287)
Supplement: Supplementary file 6 [file TableS6.DOCX]

|  | Decision 1 | Decision 2 | Decision 3 | Decision 4 | Decision 5 | Decision 6 |
| --- | --- | --- | --- | --- | --- | --- |
| Dep var: | *β* ≤ 0 | *β* ≤ 0.5 | *α* ≤ 0 | *α* ≤ 0.125 | *β* ≤ 0.25 | *α* ≤ -0.25 |
|  | (vs ≥ 0) | (vs ≥ 0.5) | (vs ≥ 0) | (vs ≥ 0.125) | (vs ≥ 0.25) | (vs ≥ -0.25) |
|  | (1) | (2) | (3) | (4) | (5) | (6) |
| CRT | -0.146* | 0.124* | 0.122* | 0.141** | 0.086 | -0.056 |
|  | (0.087) | (0.071) | (0.072) | (0.071) | (0.071) | (0.086) |
| female | 0.295 | 0.093 | -0.266 | -0.533** | 0.276 | -0.236 |
|  | (0.263) | (0.219) | (0.215) | (0.224) | (0.220) | (0.261) |
| cons | -0.900*** | 0.235 | 0.010 | 0.477* | -0.172 | -0.698** |
|  | (0.322) | (0.256) | (0.271) | (0.264) | (0.270) | (0.321) |
| ll | -465.750 | | | | | |
| Wald χ^2^ | 25.33** | | | | | |
| N | 158 | | | | | |

**Table S6. Non-egalitarian choice (option B) as a function of CRT (Study 2).** Multivariate Probit estimates. The *α* and *β* parameters associated with the dependent variable are displayed on top of each column. Robust standard errors clustered on individuals are shown in parentheses. *, **, *** denote p-values lower than 0.10, 0.05 and 0.01, respectively.
