# Supplementary material for: The cognitive basis of social behavior: cognitive reflection overrides antisocial but not always prosocial motives
Source: Front Behav Neurosci. 2015 Nov 5;9:287. doi: 10.3389/fnbeh.2015.00287 (PMC4633515; doi:10.3389/fnbeh.2015.00287)
Supplement: Supplementary file 7 [file TableS7.DOCX]

|  | Decision 1 | Decision 2 | Decision 3 | Decision 4 |
| --- | --- | --- | --- | --- |
| Dep var: | *β* ≤ 0 (vs ≥ 0) | *β* ≤ 0.5 (vs ≥ 0.5) | *α* ≤ 0 (vs ≥ 0) | *α* ≤ 0.5 (vs ≥ 0.5) |
| High CRT | -0.361 | -0.226 | 0.975*** | 0.669*** |
|  | (0.290)  [-0.078] | (0.236)  [-0.067] | (0.231)  [0.333***] | (0.238)  [0.220***] |
| female | -0.512* | -0.316 | -0.266 | -0.313 |
|  | (0.293)  [-0.110*] | (0.235)  [-0.094] | (0.223)  [-0.091] | (0.228)  [-0.103] |
| cons | -0.736*** | 0.998*** | -0.059 | 0.383** |
|  | (0.219)  [0.231] | (0.204)  [0.841] | (0.184)  [0.477] | (0.190)  [0.649] |
| Ll | -58.527 | -79.235 | -90.056 | -86.866 |
| Wald χ^2^ | 3.40 | 2.27 | 22.40*** | 12.40*** |
| pseudo R^2^ | 0.037 | 0.013 | 0.118 | 0.069 |
| N | 150 | 150 | 150 | 150 |

**Table S7. Non-egalitarian choice (option B) as a function of CRT (Study 1).** Probit estimates. High CRT is a dummy that takes value 1 if the CRT score is above the median and takes value 0 otherwise. Robust standard errors clustered on individuals are shown in parentheses and average marginal effects of the explanatory variables are shown in square brackets (for the constant, this value represents the probability obtained from normal transformation of the Probit coefficient). *, **, *** denote p-values lower than 0.10, 0.05 and 0.01, respectively.
