# Supplementary material for: The cognitive basis of social behavior: cognitive reflection overrides antisocial but not always prosocial motives
Source: Front Behav Neurosci. 2015 Nov 5;9:287. doi: 10.3389/fnbeh.2015.00287 (PMC4633515; doi:10.3389/fnbeh.2015.00287)
Supplement: Supplementary file 8 [file TableS8.DOCX]

|  | Decision 1 | Decision 2 | Decision 3 | Decision 4 | Decision 5 | Decision 6 |
| --- | --- | --- | --- | --- | --- | --- |
| Dep var: | *β* ≤ 0 | *β* ≤ 0.5 | *α* ≤ 0 | *α* ≤ 0.125 | *β* ≤ 0.25 | *α* ≤ -0.25 |
|  | (vs ≥ 0) | (vs ≥ 0.5) | (vs ≥ 0) | (vs ≥ 0.125) | (vs ≥ 0.25) | (vs ≥ -0.25) |
| High CRT | -0.585** | 0.221 | 0.460** | 0.417* | 0.178 | -0.082 |
|  | (0.281)  [-0.122**] | (0.222)  [0.073] | (0.209)  [0.172**] | (0.217)  [0.137**] | (0.208)  [0.069] | (0.254)  [-0.020] |
| female | 0.318 | -0.003 | -0.310 | -0.504** | 0.220 | -0.118 |
|  | (0.270)  [0.066] | (0.221)  [-0.001] | (0.209)  [-0.116] | (0.216)  [-0.166**] | (0.208)  [0.086] | (0.252)  [-0.029] |
| cons | -1.049*** | 0.510** | 0.149 | 0.614*** | -0.004 | -0.905*** |
|  | (0.241)  [0.147] | (0.199)  [0.695] | (0.189)  [0.559] | (0.189)  [0.730] | (0.188)  [0.498] | (0.229)  [0.183] |
| ll | -60.046 | -91.959 | -103.249 | -91.269 | -106.920 | -68.856 |
| Wald χ^2^ | 7.11** | 4.05 | 8.49** | 9.48*** | 1.52 | 0.25 |
| pseudo R^2^ | 0.058 | 0.006 | 0.041 | 0.059 | 0.007 | 0.002 |
| N | 158 | 158 | 158 | 158 | 158 | 158 |

**Table S8. Non-egalitarian choice (option B) as a function of CRT (Study 2).** Probit estimates. High CRT is a dummy that takes value 1 if the CRT score is above the median and takes value 0 otherwise. Robust standard errors clustered on individuals are shown in parentheses and average marginal effects of the explanatory variables are shown in *square brackets* (for the constant, this value represents the probability obtained from normal transformation of the Probit coefficient). *, **, *** denote p-values lower than 0.10, 0.05 and 0.01, respectively.
