# Supplementary material for: The cognitive basis of social behavior: cognitive reflection overrides antisocial but not always prosocial motives
Source: Front Behav Neurosci. 2015 Nov 5;9:287. doi: 10.3389/fnbeh.2015.00287 (PMC4633515; doi:10.3389/fnbeh.2015.00287)
Supplement: Supplementary file 9 [file TextS1.PDF]

Text S1. Order of tasks in Study 1 and Study 2

Study 1. Order of tasks
1. Short math problem
2. Adding task
[Break]
3. Social preferences elicitation task
4. Risk preferences elicitation task
[Break]
5. CRT
6. Raven

Study 2. Order of tasks
1. Self-assessment manikin (SAM test)
2. Social preferences elicitation task
3. Risk preferences elicitation task
[Break]
4. CRT
