# Supplementary figures and images for: The cognitive basis of social behavior: cognitive reflection overrides antisocial but not always prosocial motives
Source: Front Behav Neurosci. 2015 Nov 5;9:287. doi: 10.3389/fnbeh.2015.00287 (PMC4633515; doi:10.3389/fnbeh.2015.00287)

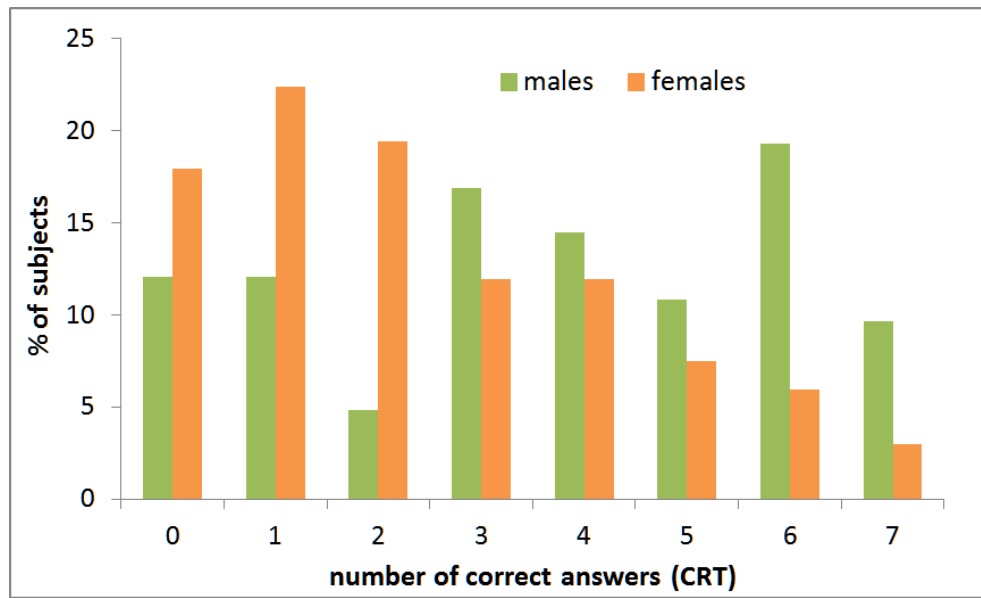

**Figure S1. Distribution of CRT scores by gender (Study 1)**

Supplement: Supplementary file 11 [file FigureS1.PDF]

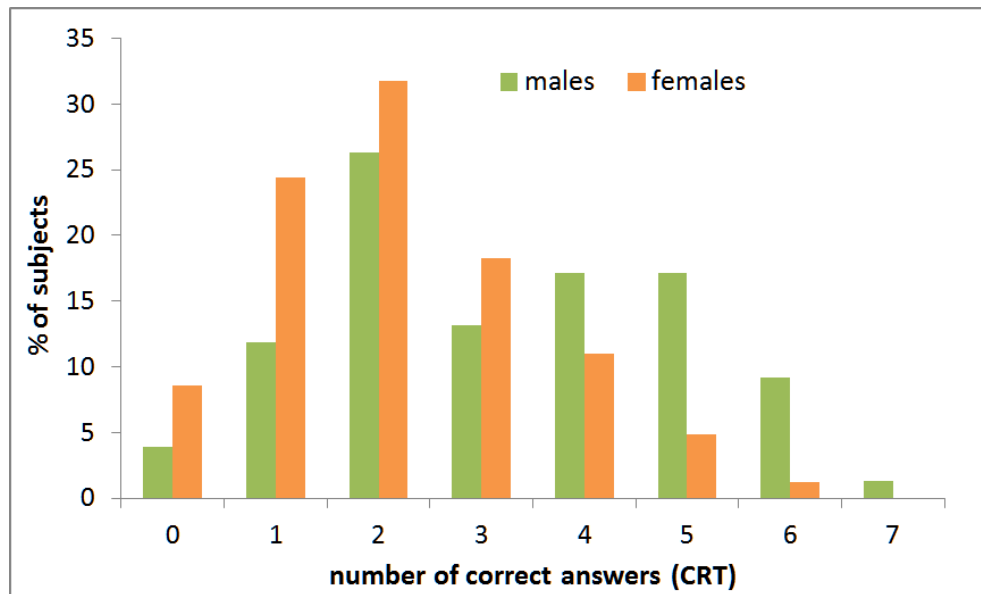

**Figure S2. Distribution of CRT scores by gender (Study 2)**

Supplement: Supplementary file 12 [file FigureS2.PDF]
